# Development and Validation of Dynamic Multi-Protein Inflammatory Scores Associated With Delirium

**DOI:** 10.1093/geroni/igaf122.3567

**Published:** 2025-12-31

**Authors:** Peter Wang, Emily Wolfson, Yi-Hsiang Hsu, Towia Libermann, Sharon Inouye, Edward Marcantonio, Long Ngo, Sarinnapha Vasunilashorn

**Affiliations:** Tufts University School of Medicine, Boston, Massachusetts, United States; Beth Israel Deaconess Medical Center, Boston, Massachusetts, United States; Marcus Institute for Aging Research, Hebrew SeniorLife, Harvard Medical School, Roslindale, Massachusetts, United States; Beth Israel Deaconess Medical Center, Boston, Massachusetts, United States; Marcus Institute for Aging Research, Hebrew SeniorLife, Harvard Medical School, Boston, Massachusetts, United States; Beth Israel Deaconess Medical Center, Boston, Massachusetts, United States; Beth Israel Deaconess Medical Center, Boston, Massachusetts, United States; Beth Israel Deaconess Medical Center, Boston, Massachusetts, United States

## Abstract

Individual inflammatory markers have been associated with delirium. However, previous work to aggregate these markers to more fully capture this joint effect did not consider correlation among the markers, nor did they yield a single inflammatory score. To address these limitations, we aimed to develop and validate preoperative (PREOP) and postoperative day 2 (POD2) inflammatory scores for delirium. We used the Successful Aging after Elective Surgery (SAGES) I study (N = 560, mean age 76.7 ± 5.2 years, 42% male) to develop these scores, and the SAGES II study (N = 420, mean age 73.3 ± 5.6, 35% male) and UK Biobank (N = 32,147, mean age 57.7 ± 8.1, 46% male) for independent validation. 6 inflammatory markers associated with delirium were considered: C-reactive protein (CRP), interleukin-6 (IL-6), soluble tumor necrosis factor alpha receptor-1 (sTNFαR1), chitinase-3 like protein-1 (CHI3L1/YKL-40), interleukin-1 receptor antagonist (IL-1RN), and lipocalin-2 (LCN-2). We used LASSO regression (outcome=delirium) to select markers for inclusion in each score. Beta coefficients from logistic regression were then employed to compute weights attributed to each marker, resulting in the following equations: PREOP score = (1/9)*log(CRP) + (5/9)*log(YKL-40) + (1/3)*log(IL-1RN) [AUC=0.684; SAGES I], validated in SAGES II (AUC=0.617) and UK Biobank (AUC=0.649); POD2 score = (1/8)*log(IL-6) + (1/5)*log(YKL-40) + (1/4)*log(sTNFαR1) + (2/5)*log(IL-1RN) [AUC=0.664; SAGES I], validated in SAGES II (AUC=0.64). In summary, the PREOP and POD2 inflammatory scores both included YKL-40 and IL-1RN. These scores may inform clinical risk assessment or future research on the interrelationship of factors such as genetics, inflammation, and delirium.

